# S28. THE ROLE OF COPING IN THE ASSOCIATION BETWEEN SUBCLINICAL PSYCHOTIC EXPERIENCES AND DAILY FUNCTIONING: EVIDENCE FROM TWO INDEPENDENT ADOLESCENT SAMPLES FROM THE GENERAL POPULATION

**DOI:** 10.1093/schbul/sby018.815

**Published:** 2018-04-01

**Authors:** Katharine Chisholm, Johanna Wigman, Danielle Hallett, Tamara Woodall, Simone Mahfouda, Renate Reniers, Eoin Killackey, Alison Yung, Stephen Wood, Ashleigh Lin

**Affiliations:** 1 University of Birmingham; 2 University of Groningen, University Medical Center Groningen, University Center for Psychiatry; 3 Telethon Kids Institute, The University of Western Australia; 4 Orygen, The National Centre of Excellence in Youth Mental Health; 5 Institute of Brain Behaviour and Mental Health, University of Manchester; 6 Telethon Kids Institute

## Abstract

**Background:**

Subclinical psychotic experiences (attenuated, brief, or limited psychotic-like experiences) are present in approximately 5% of adults and 7.5% of adolescents from the general population. Whilst the majority of these experiences are transitory, individuals who report subclinical psychotic experiences are at greater risk developing psychotic spectrum disorders, as well as other adverse outcomes. It is now well established that there is an inverse association between psychosocial functioning and (subthreshold) psychotic experiences in both clinical and non-clinical populations, however the mechanisms which drive this association are unclear. Adolescents with subclinical psychotic experiences are more likely to use maladaptive coping strategies and less likely to use adaptive ones. Given that coping styles are potentially modifiable, clarifying how coping may mediate the association between subclinical psychotic experiences and functioning could provide an important avenue for psychosocial intervention. In the current study we aimed to determine whether the association between subclinical psychotic experiences and psychosocial functioning is mediated by coping style. We conducted a within study replication in two large adolescent samples from the general populations of Australia and the United Kingdom.

**Methods:**

723 adolescents from Melbourne, Australia, and 239 adolescents from Birmingham, UK, took part in the study. Subclinical positive psychotic experiences were measured using the Community Assessment of Psychic Experiences (CAPE). The Coping Inventory for Stressful Situations (CISS) assessed three different coping styles; task oriented (adaptive) styles and emotion and avoidance oriented (maladaptive) styles. Functioning was measured via the Multidimensional Assessment of Functioning Scale (MAFS), which assesses general, family, and peer functioning. Mediation analysis was conducted using the PROCESS macro for SPSS.

**Results:**

Subclinical psychotic experiences were strongly associated with reduced general and family functioning, and to a lesser extent with reduced peer functioning. Higher subclinical psychotic experiences were associated with lower task (adaptive) and avoidance (maladaptive) oriented coping and increased emotion (maladaptive) oriented coping. Task and emotion oriented coping were found to significantly mediate the relationship between subclinical psychotic experiences and all three types of functioning in both the Melbourne and the Birmingham samples. Avoidance oriented coping was found to significantly mediate subclinical psychotic experiences and peer functioning in the Melbourne sample only. Avoidance oriented coping was not found to mediate subclinical psychotic experiences with general or peer functioning in either sample.

**Discussion:**

Given that 17% of children and 7.5% of adolescents experience subclinical psychotic experiences and that these experiences are associated with reduced functioning, high levels of distress, and suicidal ideation, introducing classroom based learning about coping strategies in schools may encourage the adoption of more positive coping strategies earlier. Additionally, the findings of the present study have important clinical treatment implications, as they suggest that techniques which increase levels of adaptive coping and reduce levels of maladaptive coping (in particular emotion-oriented styles) may help to break the cycle between subclinical psychotic experiences, functional decline, and eventual need for care.

